# Co-expression network analysis for identification of novel biomarkers of bronchopulmonary dysplasia model

**DOI:** 10.3389/fped.2022.946747

**Published:** 2022-11-07

**Authors:** Xuefei Yu, Ziyun Liu, Yuqing Pan, Xuewei Cui, Xinyi Zhao, Danni Li, Xindong Xue, Jianhua Fu

**Affiliations:** Department of Pediatrics, Shengjing Hospital of China Medical University, Shenyang, Liaoning, China

**Keywords:** brochopulmonary dysplasia (BPD), WGCNA (Weighted gene co- expression network analyses), biomarker, hub gene, hyperoxia (oxygen)

## Abstract

**Background:**

Bronchopulmonary dysplasia (BPD) is the most common neonatal chronic lung disease. However, its exact molecular pathogenesis is not understood. We aimed to identify relevant gene modules that may play crucial roles in the occurrence and development of BPD by weighted gene co-expression network analysis (WGCNA).

**Methods:**

We used RNA-Seq data of BPD and healthy control rats from our previous studies, wherein data from 30 samples was collected at days 1, 3, 7, 10, and 14. Data for preprocessing analysis included 17,613 differentially expressed genes (DEGs) with false discovery rate <0.05.

**Results:**

We grouped the highly correlated genes into 13 modules, and constructed a network of mRNA gene associations, including the 150 most associated mRNA genes in each module. *Lgals8*, *Srpra*, *Prtfdc1*, and *Thap11* were identified as the key hub genes. Enrichment analyses revealed Golgi vesicle transport, coated vesicle, actin-dependent ATPase activity and endoplasmic reticulum pathways associated with these genes involved in the pathological process of BPD in module.

**Conclusions:**

This is a study to analyze data obtained from BPD animal model at different time-points using WGCNA, to elucidate BPD-related susceptibility modules and disease-related genes.

## Introduction

Bronchopulmonary dysplasia (BPD), a chronic respiratory disease with distinctive pathological features and clinical outcomes, is common in premature infants (1). Preterm birth affects about 11% of newborns worldwide. However, due to differences in gestational age measurements, preterm birth definitions, and data collection and reporting, the incidence varies (2–4). Although the pathogenesis of BPD is not fully understood, the pathological features of alveolar dysplasia have been elucidated long-term immaturity of the alveolar structure and a reduced number of pulmonary blood vessels (5–7). Therefore, an in-depth investigation of the biomarkers and molecular mechanisms (8) involved in BPD development is required to unravel the links affecting lung development and to explore effective intervention methods.

The WGCNA algorithm is a systems biology algorithm for constructing gene co-expression networks. The algorithm is based on high-throughput gene messenger RNA (mRNA) expression data and is widely used in the international biomedical field (9). In recent years, as high-throughput biotechnology is widely used in the basic research and clinical treatment of BPD (10–12), a modular analysis tool that performs tissue/cellular networks weighted gene co-expression network analysis (WGCNA) is increasingly being used (13). First, the WGCNA algorithm assumes that the gene network obeys a scale-free distribution and defines the gene co-expression correlation matrix and the adjacency function formed by the gene network (13, 14). Second, it calculates the dissimilarity coefficients of different nodes and builds a hierarchical clustering tree accordingly (15). Different branches of the clustering tree represent different gene modules. The genes within the same modules have a high co-expression degree, while those with different scores have a low co-expression degree (16). Finally, WGCNA reveals the relationship between the modules and specific phenotypes or diseases to identify target genes and gene networks (9); the analysis provides system-level insight and high sensitivity for genes with low abundance or small folding changes, without any information loss (17, 18).

In this study, through comprehensive bioinformatics analysis, we analyzed the RNA-Seq data from our previous study to identify differentially expressed genes (DEGs) between BPD and control rat models to elucidate the mechanism behind BPD pathogenesis. Therefore, in this study we elucidated genes that they may be used as diagnostic indicators and therapeutic markers for BPD.

## Materials and methods

### Animal model

According to previous research methods, long-term hyperoxia exposure after newborn has been considered to be a relatively complete method in animal models that can delay alveolar development in previous studies (6, 19, 20). Newborn Sprague–Dawley rats were randomly and double-blindly divided into a control group and a model group. Following previous method (20–22), confounding factors such as birth weight, sex, gestational age, and temperature were excluded by random assignment. The light/dark cycle was 12 h, and the rat had free access to food and water. The control group was fed in the air (FiO_2 _= 0.21), and the model group was placed in a hyperoxia box to maintain the oxygen concentration in the box (FiO_2 _= 0.85). Lime soda was placed in oxygen box to absorb CO_2_ less than 0.5%. Silica gel was used to remove the water vapor in the oxygen tank so that the humidity in the box was maintained at 60%–70%, and the temperature of the two groups was maintained at 25–26 °C. The dams were fed neonatal rats and were shuffled between different cages every 24 h to reduce the effect of differences in lactation ability. The cages were opened for 30 min daily, and the rats were provided with clean drinking water and food. On days 1, 3, 7, 10, and 14 after birth, the neonatal pups were randomly selected from each group. After administration of anesthesia (isoflurane *via* inhalation), the thoracic cavity of the rat was opened, the lung tissue was lavaged with 18 cm H_2_O pressure normal saline, and the blood in the lung was perfused. The lung lobes of were separated and placed in tubes. After quick freezing in liquid nitrogen, the lung samples were stored at −80 °C for RNA-Seq. The procedures and design of the experiments involving animals complied with the guidelines for animal care and use. This study was approved by the Ethics Committee of China Medical University (ethics code: 2020PS764K).

### Data preprocessing

The RNA-seq data of this study is uploaded into the GEO database (https://www.ncbi.nlm.nih.gov/geo/query/acc.cgi). The Gene Expression Omnibus (GEO) accession number is GSE 212098. We also obtained the BPD-related transcriptomic dataset GSE156028 from GEO. They analyzed tracheal aspirates (TAs) from 53 neonates receiving invasive mechanical ventilation. 26 infants were diagnosed with extremely preterm birth without lung disease, and 27 term infants received invasive mechanical ventilation for elective surgery. The “affy” package in R was used for normalization and background correction of the data. The probe-level data were then converted to gene expression values. For multiple probes corresponding to a gene, the average expression value was taken as the gene expression value in this study. We observed the distribution patterns of disease and control samples (before and after cluster analysis and outlier removal) using principal component analysis (PCA).

### Identification of DEGs

The “limma” package in R was used to identify DEGs between the expression data of BPD and healthy control samples. We performed a significance analysis on the transcriptomic mRNA profiles data and set the selection criteria as false discovery rate (FDR) values <0.05 and |log_2_ Fold change| > 0 for network construction.

### Construction of co-expression network

We analyzed the connectivity of eigengenes. Eigengenes can provide information about the relationship between pairs of gene co-expression modules. The “WGCNA” package in R was used to construct co-expression networks based on the expression data profiles. We constructed a network of mRNA gene associations, including the 150 most associated mRNA genes in each module, using the STRING database and Cytoscape software to construct a protein-protein interaction (PPI) network. Transcriptomic mRNA profiles data quality was checked by the “impute” package in R, which detects genes for missing values and ensures that they are good samples. We performed sample clustering to draw sample trees, and detect and remove outliers. We then constructed a Pearson correlation matrix for paired genes and found a soft threshold power *β* value by using the pickSoftThreshold function of the “WGCNA” package.

### Identification of hub genes

We defined hub genes in the co-expression network as genes that satisfy two conditions: the absolute value of Pearson correlation for module membership (MM) > 0.8 and the absolute value of Pearson correlation for gene-trait (GS) relationship > 0.2, representing high modular connectivity and high clinical significance. The true central genes were subsequently obtained by obtaining the intersection of the central genes and significant DEGs in a co-expression network visualized by Cytoscape 3.7.1.

### Enrichment analysis

Based on the results in the previous section, we focused on modules that were differentially expressed consistently across developmental time. We performed GO and KEGG pathway enrichment annotations for candidate genes in up-regulated brown modules and down-regulated purple modules at all time points in the BPD model. The online software DAVID (Database for Annotation, Visualization, and Integrated Discovery v6.8; https://david.ncifcrf.gov/) was used to investigate gene-related biological processes and pathways. GO biological processes and KEGG pathways with *P* < 0.05 and/or FDR < 0.05 were defined as significant terms. GO terms are divided into biological process (BP), cellular component (CC), and molecular function (MF). Statistical significance was considered at *P*-value < 0.05.

## Results

### Construction of WGCNA and selection of soft threshold

**Figure 1:**
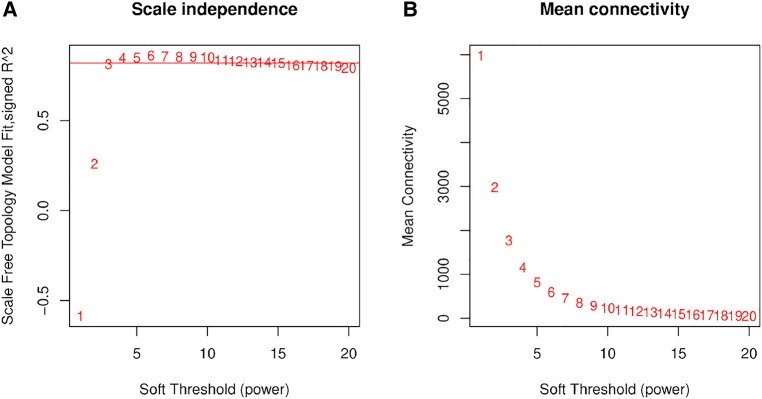
Network topology analysis of soft threshold power. (**A**) The *x*-axis reflects soft threshold capability, and the *y*-axis reflects the scale-free topological model fit index. (**B**) The *x*-axis reflects the soft threshold capability, and the *y*-axis reflects the average connectivity (degrees).

We used RNA-Seq data of normal controls (*n* = 30, 3 repetitions per set from postnatal days 1, 3, 7, 10, and 14) and BPD rat models (*n* = 30, 3 repetitions per set from postnatal days 1, 3, 7, 10, and 14) for analysis. We examined genes and samples with several missing values, but all genes passed the cutoff threshold. Next, we calculated the correlation coefficient (Pearson Coefficient) between any two genes of a sample and used the weighted value of the correlation coefficient to make the connection between the genes in the network obey scale-free networks. In this study, the weight parameter *β* was 3. Since the scale independence reached 0.8, the data had a relatively high average connectivity (Figure 1).

### Module hierarchical clustering and correlation analysis

**Figure 2:**
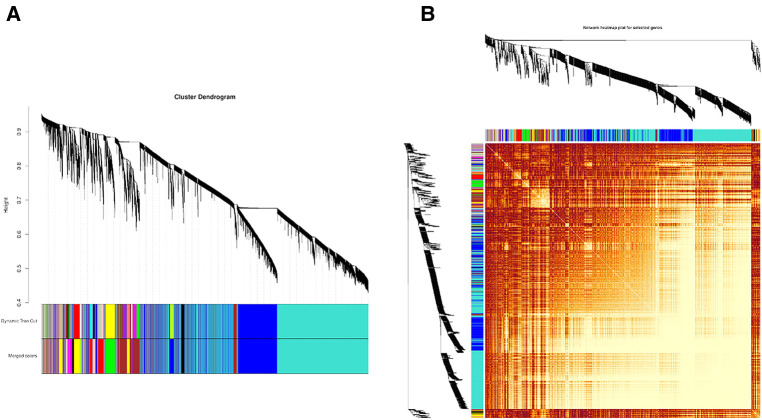
(**A**) Module hierarchical clustering tree. The upper part of the tree diagram, the vertical distance represents the distance between two nodes (genes). (**B**) Heatmap representing the clustering of all modules (Maps represent the relationship between the identified modules). The heat map depicts the topological overlap matrix (TOM) between all genes included in the analysis. Light colors indicate low overlap, and progressively darker red indicates high overlap.

**Figure 3:**
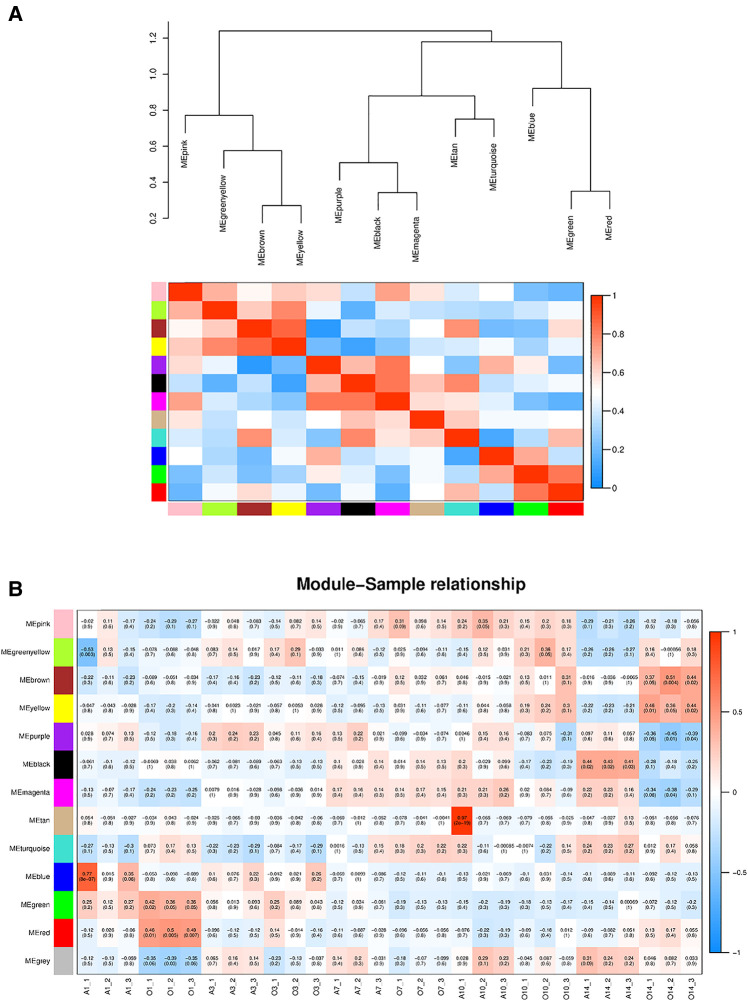
(**A**) Heatmap of the correlation between modules from the gene dendrogram and the self-gene adjacency graph (the upper part clusters the modules according to the eigengenes of the modules. The ordinate represents the degree of dissimilarity of the nodes; each row in the lower part of the graph. The sum column represents a module). (**B**) Module-feature associations (Each row corresponds to a module, and each column corresponds to a sample. Each cell contains the corresponding correlation and *P*-value. The table is color-coded by correlation according to the color legend).

First, the distance between samples was calculated using an algorithm describing the community's composition and structure, i.e., hierarchical clustering analysis was performed according to the beta diversity distance matrix, and 13 gene co-expression modules were finally constructed. The topological overlap matrix (TOM) between all genes included in the analysis is depicted by a heatmap. Light colors indicate low overlap, and progressively darker reds indicate high overlap. The results showed strong gene expression connectivity between modules (Figure 2). The results revealed that the 13 modules could be grouped into two clusters in each sample at different time points. (Figure 3), and four combinations (module brown and yellow, module black and magenta, module tan and turquoise, and module green and red) were highly interactive. We associated modules with features and searched for the most significant associations. The results showed that module red had the most significant positive correlation with BPD model at day 1; the module brown and yellow had the most significant positive correlation with BPD model at day 14, and the module purple had the most significant negative correlation with BPD model at day 14 (Figure 3).

### Gene expression patterns of each module

**Figure 4:**
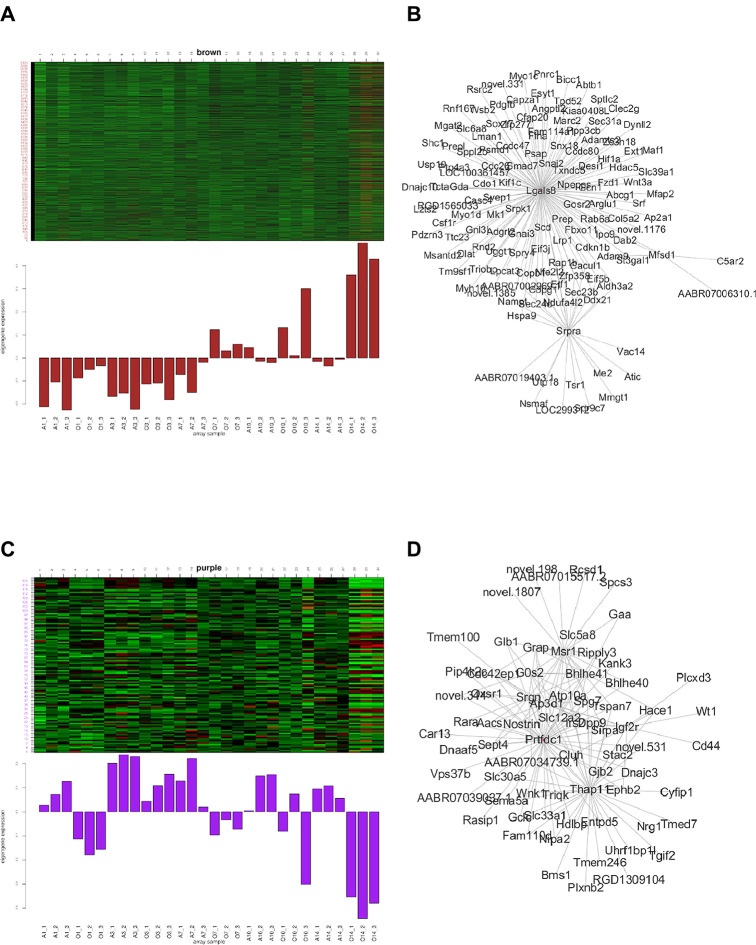
Gene expression pattern of each module (the left part exhibits the gene expression pattern, and the right part shows the hub-gene expression network).

Both in the green module and yellow module, the expression pattern of the BPD group was up-regulated at days 10 and 14. *Rassf5* etc. were the most related hub genes of this module. In the black module, the expression pattern of the BPD group was up-regulated at day 1 and significantly down-regulated at days 10 and 14. In the yellow module, the expression pattern of the BPD group was down-regulated at day 1, and significantly up-regulated at days 10 and 14; PPI network revealed *Prickle4* and *Sod2* as the most significant hub genes in this module. In the magenta module, the expression pattern of the BPD group was down-regulated at days 10 and 14; PPI network analysis revealed *Fgfr4*, *Jak3*, and *Tcf7* as the most significant hub genes in this module. In the red module, the expression pattern of the BPD model group was up-regulated at day 1; PPI network analysis revealed *Dusp4*, *Golm1*, and *Kif16b* as the most significant hub genes in this module. In the turquoise module, the expression pattern of the BPD model group was up-regulated at days 1 and 7, and down-regulated at day 14; PPI network analysis revealed *Dctn4*, *Cox4i1*, and *Ppib* as the most significant hub genes for this module (see Figures in Supplementary Materials). In the brown module, the expression pattern of the BPD group was up-regulated at days 1, 3, 7, 10, and 14; PPI network analysis revealed *Lgals8* and *Srpra* as the hub genes of this module. In the purple module, the expression pattern of the BPD model group was down-regulated at days 1, 3, 7, 10, and 14; PPI network analysis revealed *Prtfdc1* and *Thap11* as the hub genes of this module (Figure 4).

### Functional annotation of hub genes in candidate modules

**Figure 5:**
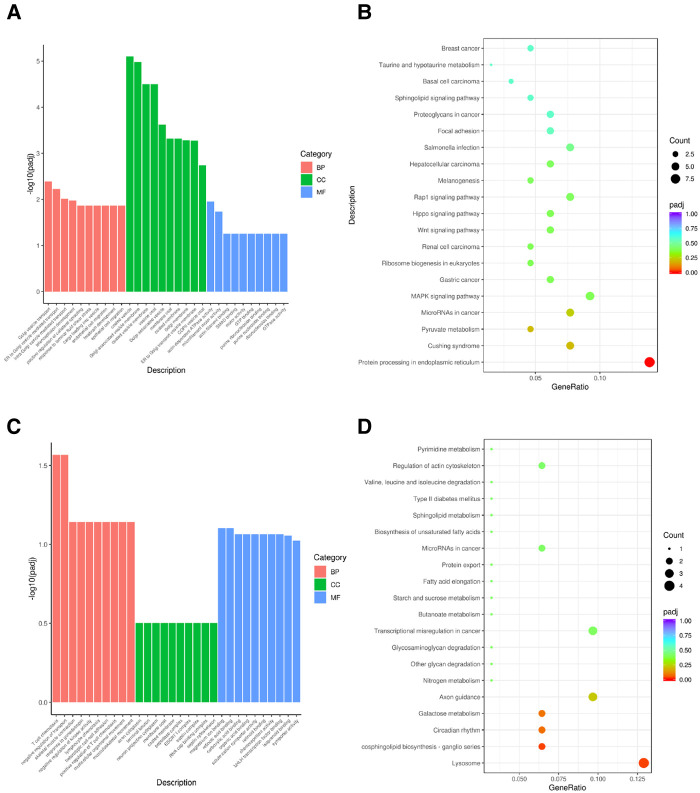
KEGG and GO pathway annotations of candidate module genes (**A**) GO enrichment of brown module; (**B**) KEGG enrichment of brown module; (**C**) GO enrichment of purple module; (**D**) KEGG enrichment of purple module.

We performed GO and KEGG pathway enrichment annotations for candidate genes in brown modules that were up-regulated and in purple modules that were down-regulated at all time-points in the BPD model. The up-regulated genes of the brown module were mainly enriched in the following GO terms: Golgi vesicle transport (BP), coated vesicle (CC), and actin-dependent ATPase activity (MF) pathways; KEGG enrichment analysis revealed that they were mainly enriched in the protein processing in endoplasmic reticulum pathway. The down-regulated genes of the purple module were mainly enriched in the following GO terms: T cell chemotaxis (BP), axon cytoplasm (CC), and magnesium ion binding pathways; KEGG enrichment analysis revealed that they were mainly enriched in the lysosome pathway (Figure 5).

### Differential expressions were verified in clinical patients samples

**Figure 6:**
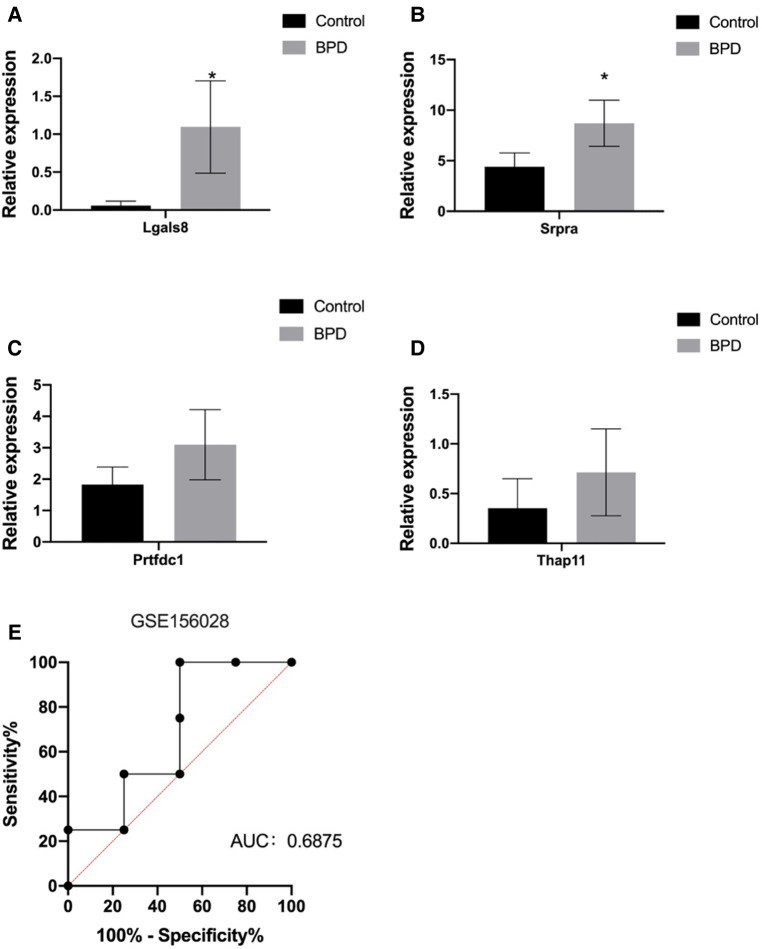
(**A–D**) Validation of expression levels of Lgals8, Srpra, Prtfdc1, and Thap1 in tracheal aspirate samples from normal controls and BPD patients (*n* = 38) from GSE156028 in the gene expression omnibus (GEO) database. (**E**) Receiver operating characteristic curve for diagnosis of BPD patients using expression levels of these four RNAs in GSE156028. Data are shown as the mean ± SEM. *P < 0.05.

Finally, we validated the differentially expressed differentially identified genes of interest with RNA-Seq data from clinical patient samples from the Gene Expression Omnibus (GEO) database. GSE156028 was selected as tracheal aspirate samples from normal controls and patients with BPD (*n* = 38). The results showed that the expression of Lgals8, Srpra, Prtfdc1, and Thap11 were up-regulated in BPD, and the difference of Lgals8 and Srpra was statistically significant (Figure 6). The diagnostic effect was judged according to the sensitivity, specificity and area under the receiver operating characteristic (ROC) curve. The AUC value was 0.6875, which has certain diagnostic significance. It is expected to continue to be verified in the expanded clinical database samples in the future.

## Discussion

BPD is a multifactorial disease that remains a major therapeutic challenge in neonatal care (23, 24). According to a study conducted by the Neonatal Research Network of the National Institutes of Child Health and Human Development, the overall incidence of BPD in infants born at less than 28 weeks of gestation is estimated to be between 48% and 68% (4). In this study, we used the complete RNA-Seq data of our research group to determine the key features of the BPD rat model at different time-points by constructing WGCNA modules, using data from lung tissue of rat BPD model, and comparing the data from model group with those from the control group. Key genes, including Lgals8, Srpra, Prtfdc1, Thap11, were identified.

In the brown module, the expression pattern of the BPD group was up-regulated at days 1, 3, 7, 10, and 14; PPI network analysis revealed Lgals8 and Srpra as the hub genes of this module. Lgals8 (Lectin, galactoside-binding soluble 8) codes for a member of the galactose family. Galectins are galactoside-binding animal lectins with a conserved carbohydrate recognition domain. Galectins have important functions in development, differentiation, cell adhesion, cell-matrix interactions, growth regulation, apoptosis, and RNA splicing (25). The gene is widely expressed in tumor tissues and is involved in integrin-like cell interactions. Additionally, spliced transcript variants of the gene encoding different isoforms have been identified. It is also commonly expressed in 27 tissues including the spleen, skin, and lung (26–28). Srpra (SRP receptor subunit alpha) is a subunit of the endoplasmic reticulum signal-recognition granule receptor, which, together with the signal-recognition granule, is involved in the targeting and translocation of secreted and membrane proteins marked by the endoplasmic reticulum signal sequence. Alternative splicing results in multiple transcript variants (29–32). Therefore, it may play an important role in the process of lung development and the pathological process of BPD.

In the purple module, the expression pattern of the BPD model group was down-regulated at days 1, 3, 7, 10, and 14; PPI network analysis revealed *Prtfdc1* and *Thap11* as the hub genes of this module. Prtfdc1 (phosphoribosyl transferase domain containing 1) is a hypoxanthine-guanine phosphoribosyltransferase (PRT) homolog of unknown function that catalyzes the conversion of hypoxanthine and guanine to their respective monophosphate-enabled proteins homodimerization activity, and is involved in purine ribonucleoside rescue (33, 34). Thap11 (Thanatos-associated protein domain containing protein 11) is a ubiquitously-expressed member of the transcription factor family with a highly conserved DNA-binding and protein-interacting region (35), involved in various cellular regulations such as apoptosis, proliferation and differentiation (36–38). Relevant studies of the above genes in BPD are lacking, and their role in BPD models deserve further research.

Functional enrichment analysis revealed that the hub genes of the brown module were mainly enriched in Golgi vesicle transport (BP), coated vesicle (CC), and actin-dependent ATPase activity (MF) pathways. KEGG enrichment analysis revealed that these genes were mainly enriched in protein processing in endoplasmic reticulum pathway. These results are in accordance with those from our previous study on the pathogenesis of BPD, which suggested that cellular lipid membrane activities, such as those of the endoplasmic reticulum, were involved in the pathological process of BPD (39), and many studies also confirmed that pathways, such as YAP/TAZ mediate actin-dependent ATPase and endoplasmic reticulum function pathways (40–42), which are involved in the functional changes of alveolar epithelial cells. These studies confirmed that these pathways were involved in BPD pathogenesis. The GO enrichment analysis of the down-regulated hub genes of the purple module revealed that the following terms were enriched: T cell chemotaxis (BP), axon cytoplasm (CC) and magnesium ion binding pathways. KEGG enrichment analysis revealed that the genes were mainly enriched in pathways such as the lysosome pathway. Concurrently, previous studies also showed that T cell receptors are reduced in BPD (43) and may be a risk factor for infection (44). Finally, the tracheal aspirate samples of BPD patients in the GEO database were used to verify *Lgals8*, *Srpra*, *Prtfdc1*, and *Thap11* expression, and the results showed that the above genes were up-regulated in BPD.

Significant impairment of lung development results in persistent airway and pulmonary vascular disease, which affects adult lung function (45–48). Clinical and translational research that improve phenotypic classification of BPD and enable early identification of at-risk preterm infants should improve trial design and individualized care, which can improve outcomes for preterm infants (49). In this study, we cited the results of Oji Mmuo et al*.* (50), they analyzed the transcriptome characteristics of tracheal aspirates in extremely preterm infants with BPD and term infants without BPD. BPD is a complex disease with multiple contributing factors, including genetic predisposition, epigenetic factors, arrest of lung development, chronic inflammation, mechanical ventilation, and oxygen toxicity. In this study, we focused on the simplified lung structure of premature infants caused by long-term hyperoxia exposure and the normal premature infant model from different time points. We explored the transcriptome characteristics in the development of BPD. Oji-Mmuo et al. (51) also analyzed miRNA and mRNA profiles in tracheal aspirates, and due to the small number of clinical samples, the expression of further large-scale clinical studies deserves attention. It is believed that in the near future, the research on BPD markers will have important progress.

## Conclusion

This study revealed the potential biological targets and enrichment pathways of BPD through the WGCNA analysis method, which paves the way for future BPD research and early clinical diagnosis and treatment of BPD. This is the first study that integrates data obtained from animal models of BPD at different time-points to construct co-expression networks using the WGCNA approach to explore BPD-related susceptibility modules and disease-related genes. Our findings reveal abnormal modules and several key genes that enhance our fundamental understanding of the molecular mechanisms of BPD.

## Data Availability

The raw data supporting the conclusions of this article will be made available by the authors, without undue reservation.
